# National Latino AIDS Awareness Day — October 15, 2014

**Published:** 2014-10-10

National Latino AIDS Awareness Day is observed each year on October 15 to focus on the continuing and disproportionate effects of human immunodeficiency virus (HIV) infection and acquired immune deficiency syndrome (AIDS) on the Hispanic or Latino population in the United States. Two of the three goals of the National HIV/AIDS Strategy are to reduce HIV incidence and to reduce HIV-related disparities (1).

Estimates of HIV incidence for 2010 indicate that Hispanics or Latinos had a rate of 27.5 per 100,000 population compared with 8.7 for non-Hispanic or Latino whites (2). In 2010, male-to-male sexual contact was attributed to an estimated 68% of new infections among all Hispanics or Latinos and an estimated 79% of new infections among Hispanic or Latino males. Among Hispanic or Latino females, high-risk heterosexual contact was attributed to an estimated 86% of new infections. Data from CDC’s National HIV Behavioral Surveillance System show that, in 2011, 37% of Hispanic or Latino men who have sex with men did not know they were infected compared with 14% of non-Hispanic or Latino white men who have sex with men (3).

National Latino AIDS Awareness Day is an opportunity to encourage increased HIV prevention activities, such as HIV testing, for Hispanics or Latinos. CDC supports testing, access to care and treatment, and a range of other efforts to reduce HIV infection among Hispanics or Latinos. Additional information about CDC resources and activities for National Latino AIDS Awareness Day is available at http://www.cdc.gov/hiv/risk/racialethnic/hispaniclatinos.

## References

[1] (2010). The National HIV/AIDS Strategy for the United States and the National HIV/AIDS Strategy: federal implementation plan.

[2] CDC Estimated HIV incidence in the United States, 2007–2010. HIV surveillance supplemental report 2012.

[3] Wejnert C, Le B, Rose CE (2013). HIV infection and awareness among men who have sex with men—20 cities, United States, 2008 and 2011. PLoS One.

